# Global Warming Attenuates the Tropical Atlantic-Pacific Teleconnection

**DOI:** 10.1038/srep20078

**Published:** 2016-02-03

**Authors:** Fan Jia, Lixin Wu, Bolan Gan, Wenju Cai

**Affiliations:** 1 Institute of Oceanology, Chinese Academy of Sciences, and Key Laboratory of Ocean Circulation and Wave, Chinese Academy of Sciences, Qingdao, China; 2 Physical Oceanography Laboratory/CIMST, Ocean University of China and Laboratory for Ocean Dynamic Process and Climate, Qingdao National Laboratory for Marine Science and Technology, Qingdao, China; 3 CSIRO Oceans and Atmosphere Flagship, Aspendale, Victoria 3195, Australia

## Abstract

Changes in global sea surface temperature (SST) since the end of last century display a pattern of widespread warming intercepted by cooling in the eastern equatorial Pacific and western coasts of the American continent. Studies have suggested that the cooling in the eastern equatorial Pacific may be partly induced by warming in the North Atlantic. However, it remains unknown how stable this inter-tropical teleconnection will be under global warming. Here we show that the inter-tropical teleconnection from the tropical Atlantic to Pacific weakens substantially as the CO_2_ concentration increases. This reduced impact is related to the El Niño-like warming of the tropical Pacific mean state, which leads to limited seasonal migration of the Pacific inter-tropical convergence zone (ITCZ) and weakened ocean heat transport. A fast decay of the tropical Atlantic SST anomalies in a warmer climate also contributes to the weakened teleconnection. Our study suggests that as greenhouse warming continues, the trend in the tropical Pacific as well as the development of ENSO will be less frequently interrupted by the Atlantic because of this attenuation. The weakened teleconnection is also supported by CMIP5 models, although only a few of these models can capture this inter-tropical teleconnection.

SST variations over the tropical Pacific can exert great impacts on the regional and global climate^1^. Several studies have suggested that the recent cooling over the tropical Pacific contributed to the slowdown of global surface temperature warming since the end of last century^2^,^3^,^4^. The cooling in the tropical Pacific SST has multiple origins including internal coupled ocean-atmosphere interaction, atmospheric stochastic noise, and other external forcings^3^,^5^,^6^.

While the tropical Pacific plays a dominant role in driving global tropical SST variations, evidence suggests that there is a close connection between the north tropical Atlantic (NTA) and El Niño Southern Oscillation (ENSO) events^7^,^8^,^9^,^10^,^11^. On the one hand, NTA warm SST anomalies can emerge after the peak of an El Niño event^12^,^13^. On the other hand, NTA warm SST anomalies during boreal spring can lead to a La Niña-like tropical Pacific response in the following winter. More specifically, an NTA warm SST anomaly in boreal spring can induce an inter-hemispheric SST dipole over the eastern tropical Pacific through a coupled wind-evaporative-SST (WES) feedback^14^. Due to the seasonal migration of the ITCZ and the positive Bjerknes feedback, the eastern tropical Pacific dipole subsequently evolves into an ENSO-like pattern^8^,^9^.

Recent studies have shown that the decadal Atlantic warming may explain part of the Walker circulation strengthening during 1990–2012 and amplify the cooling trend over the tropical Pacific^15^,^16^,^17^,^18^. Since the teleconnection from the tropical Atlantic to the Pacific dominates at interannual timescales^7^,^8^,^9^,^10^,^11^, the decadal impacts could be interpreted as the net effect over a period of time of these individual interannual events. While the interannual teleconnection is robust over the twentieth century (Fig. 1), it remains unclear whether this teleconnection will be modulated by greenhouse warming. This is an important question because it determines whether the North Atlantic’s strong uptake of excessive heat caused by elevation of greenhouse gases^19^ will be able to continue modulating the SST warming trend in the tropical Pacific, as seen in the recent decades.

Given the complexity of fully coupled ocean-atmosphere general circulation models (CGCMs), it is difficult to decipher effects of different ocean and atmosphere dynamic processes on the inter-basin interactions. This motivates us to use an intermediate climate model with a full atmospheric GCM coupled to a reduced-gravity ocean (RGO) model. The coupled model consists of a Community Atmosphere GCM, version 3.1 (CAM3.1) and a Zebiak-Cane type 1.5-layer RGO model (the CAM3.1-RGO model hereafter; Supplementary Information).

## Results

### Model performance

The model produces realistic tropical climatology and ENSO properties (Supplementary Figs S1and S2), more so than most of the fully coupled GCMs^20^. These realistic features include the position of the warm pool and cold tongue in the tropical Atlantic and Pacific, and ENSO amplitude and frequency that are comparable to the observed. The modeled marine ITCZ that is situated near the equator in December-February (DJF; Fig. S1a) and moves northward to ~10 °N in June-August (JJA; Fig. S1b) over the tropical Pacific and north of the equator in both seasons over the tropical Atlantic is also reproduced.

**Figure 1:**
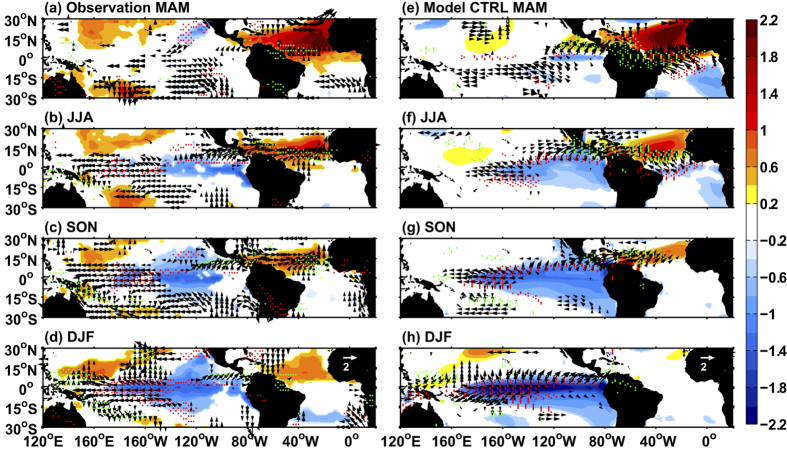
Observed and modeled Atlantic to Pacific inter-tropical teleconnection. Maps showing development of the teleconnection obtained by regressing seasonal SST anomalies onto NTA SST (90°W–20°E, 0–25°N) averaged over the January-March (JFM) season (shaded), wind vectors at 10m (m s^−1^ °C^−1^; vectors) and precipitation (red and green dots indicate negative precipitation greater than 1 mm d^−1^ °C^−1^ and positive precipitation greater than 1 mm d^−1^ °C^−1^, respectively) during the MAM (**a,e**), JJA (**b,f**), SON (**c,g**) and DJF (**d,h**) season. The left (**a–d**) and right (**e–h**) columns are derived from observations (HadISST for SST and 20CRv2 for wind and precipitation, covering the 1900–2012 period) and model CTRL (100 year at equilibrium state), respectively. Only values above the 95% confidence level are shown. All the maps were generated in MATLAB.

The model generates the observed teleconnection from the NTA to the tropical Pacific (Fig. 1). In boreal spring (March-May, MAM), a positive NTA SST anomaly creates a surface low in the western tropical Atlantic, which induces a wetter-than-average condition over Central America and westerly wind anomalies over the northeastern tropical Pacific (Fig. 1a,e). The anomalous westerlies decelerate the northeast trades, leading to a reduction of evaporative cooling and thus warming of the ocean. In the model, this warm SST anomaly north of the equator could set up an anomalous southward pressure gradient, triggering the WES feedback and resulting in an inter-hemispheric SST dipole (Fig. 1e). However, SST responses in the observational results are less obvious over the eastern tropical Pacific (Fig. 1a), which may result from the balance of diverse oceanic and atmospheric processes.

As the ITCZ moves northward in boreal summer (JJA), the southeast trades dominate in the northeastern tropical Pacific, which favors a northward extension of the negative SST through enhanced evaporative cooling and vertical advection (Fig. 1b,f). In the following seasons, the cold SST anomaly is further amplified due to oceanic advections, and propagates to the west through the coupled ocean and atmosphere processes as indicated by easterly wind anomalies emerging over the western part of the cooling. A La Niña-like pattern is eventually developed in boreal winter (DJF), with cold SST anomalies and decreased rainfall located over the central and eastern tropical Pacific (Fig. 1d,h).

### Weakened tropical Atlantic-Pacific teleconnection under greenhouse warming

To examine the response of the tropical Pacific to the Atlantic forcing under different CO_2_ concentrations, we conduct two simulations (Methods), including a 400-yr doubled CO_2_ concentration experiment (2CO_2_) and a 400-yr quadrupled CO_2_ concentration run (4CO_2_). The change in the tropical Pacific mean state displays an El Niño-like pattern, with strong SST warming in the eastern tropical Pacific by 1.2 °C and 3 °C (Supplementary Fig. S3), respectively. In association with that, the Walker circulation and the tropical Pacific ocean currents weaken (Supplementary Fig. S4).

**Figure 2:**
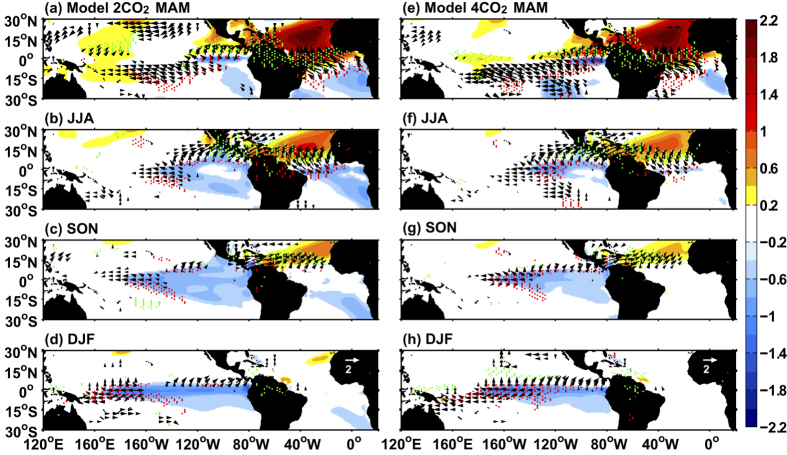
Modeled Atlantic to Pacific inter-tropical teleconnection under greenhouse warming. Maps showing development of the teleconnection obtained by regressing seasonal SST anomalies onto NTA SST (90°W–20°E, 0–25°N) averaged over the January-March (JFM) season (shaded), wind vectors at 10m (m s^−1^ °C^−1^; vectors) and precipitation (red and green dots indicate negative precipitation greater than 1 mm d^−1^ °C^−1^ and positive precipitation greater than 1 mm d^−1^ °C^−1^, respectively) during the MAM (**a,e**), JJA (**b,f**), SON (**c,g**) and DJF (**d,h**) season. The left (**a–d**) and right (**e–h**) columns are derived from the equilibrium state (last 100 years) of 2CO_2_ and 4CO_2_, respectively. Only values above the 95% confidence level are shown. All the maps were generated in MATLAB.

The increased CO_2_ leads to a weakening of the tropical Pacific response to the tropical Atlantic (Fig. 2). A similar NTA warming of ~2 °C during boreal spring causes ~1.8 °C cooling in the tropical Pacific during boreal winter in CTRL (Fig. 1h), which decreases to ~1 °C in 2CO_2_ (Fig. 2d) and ~0.5 °C in 4CO_2_ (Fig. 2h). Further, the La Niña-like cooling with increased CO_2_ concentration is more equatorially confined to 2°S–2°N.

To further substantiate the above regression analysis, three sets of initial-value ensemble runs are carried out (hereafter, we call them IV4-CTRL, IV4-2CO_2_, IV4-4CO_2_, respectively). In each member, a uniform SST anomaly with amplitude of +4 °C is initiated over the NTA (0–25°N) region on the first day of January and maintained for two years within a fully coupled environment. A total of 30 member ensemble experiments are then performed for every set, each starting from a slightly different initial condition based on CTRL, 2CO_2_ and 4CO_2_, respectively (Methods).

**Figure 3:**
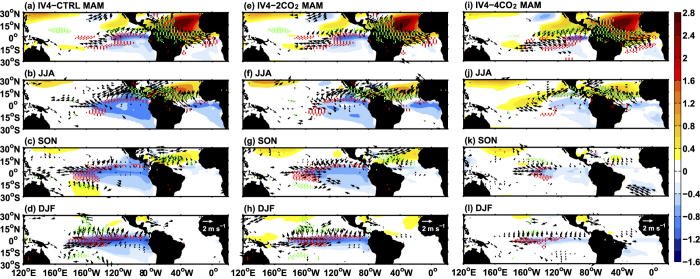
Response of the tropical Pacific to NTA warming under elevated CO_2_. Ensemble-and seasonal-mean changes in SST (°C; shaded), wind vectors at 10m (m s^−1^; vectors) and precipitation (red and green dots indicate negative precipitation greater than −1 mm d^−1^ and positive precipitation greater than 1 mm d^−1^, respectively) during the MAM (**a,e,i**), JJA (**b,f,j**), SON (**c,g,k**) and DJF (**d,h,l**) seasons. The left (**a–d**), middle (**e–h**) and right **(i–l**) columns denote changes in IV4-CTRL, IV4-2CO_2_ and IV4-4CO_2_ compared with the corresponding IV0 runs, respectively. Only values above the 95% confidence level are shown. All the maps were generated in MATLAB.

The sensitivity experiments reproduce the teleconnection from the NTA to the tropical Pacific as well as its weakening under a higher CO_2_ forcing (Fig. 3). The development processes of the cold SST anomalies after boreal spring weaken markedly in IV4-2CO_2_ and IV4-4CO_2,_ compared with those in IV4-CTRL. The amplitude of the tropical Pacific cooling in boreal winter reduces from ~ 1 °C in IV4-2CO_2_ (Fig. 3h), to ~0.5 °C in IV4-4CO_2_ (Fig. 3l), which is 66% and 33% of that in IV4-CTRL, respectively. To better understand the potential mechanisms, we analyze the changes season by season.

### Mechanism

In boreal spring, SST dipoles with a similar pattern and intensity can be found over the eastern tropical Pacific in the three scenarios (Fig. 3a,e,i). As discussed above, the SST dipole is mainly induced by the WES feedback, indicating an important role of latent heat flux (LHF) in its formation. Based on the standard bulk formula (Supplementary Information), LHF can be decomposed into atmospheric forcing because of changes in wind speed and oceanic response because of evaporation (that is, 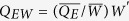
and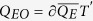; where the overbar denotes time mean, the prime denotes departure from the mean, *W* is the surface wind speed, 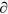 is a constant and *T* is SST). We diagnose the two effects over the northern pole of the SST dipole (5°N–20°N, 140°W–95°W) and find that the evaporative cooling (*Q*_*EO*_) is stronger in higher CO_2_ scenarios as a result of warmer background SST (Supplementary Fig. S5). At the same time, the warming effect caused by the decreased wind (*Q*_*EW*_) is greater in IV4-2CO_2_ and IV4-4CO_2_, even if the wind speed changes are similar (~1 m s^−1^). Recall that a stronger El Niño-like response will form under a higher CO_2_ forcing over the tropical Pacific, associated with a weaker background easterly (Supplementary Fig. S4, UW), and thus enhanced atmospheric forcing. Overall, the combined effects (*Q*_*EO*_ + *Q*_*EW*_) remain the same, resulting in a similar SST dipole between the three sets of experiments.

In boreal summer, the southern pole of the SST dipole extends to the north with smaller area and amplitude in higher CO_2_ scenarios (Fig. 3b,f,j). The smaller cold SST anomaly in the eastern tropical Pacific induces smaller changes in the zonal SST gradient, associated with a weaker easterly wind anomaly. Because the mean position of the ITCZ is located further equatorward with increasing CO_2_ (~6°N in CTRL, ~5.1°N in 2CO_2_ and ~3.3°N in 4CO_2_ based on the latitude of maximum precipitation), the summer northward migration of the ITCZ is limited, as is the extension of cold SST anomalies to the northeastern tropical Pacific. Moreover, the El Niño-like warming can diminish the zonal and meridional SST gradients of the tropical Pacific (Supplementary Fig. S4, d_x_SST and d_y_SST), weakening the ocean dynamic cooling through decreased advection of background temperature by anomalous currents.

**Figure 4:**
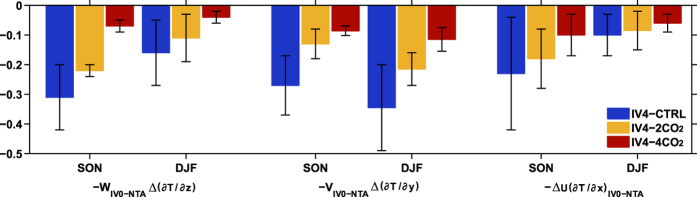
Dominant ocean heat transport in the tropical Pacific under increased CO_2_ forcing. Ensemble-mean changes of ocean heat transport components (unit: 1 × 10^−7^ °C s^−1^) during SON and DJF over the central-eastern Pacific (10°S–10°N, 180°–90°W). The bars denote changes of the IV4-CTRL (blue), IV4-2CO_2_ (orange) and IV4-4CO_2_ (red) from the corresponding IV0 runs (see text for more details about the experiments). Standard deviation bars based on the standard deviation of ensemble members are also shown.

After boreal summer, the cold SST anomalies in the eastern tropical Pacific gradually develop into La Niña-like patterns through the ocean dynamic processes. To examine which terms of the ocean heat transport dominate in the ensuing response, we apply a mixed layer heat budget (Supplementary Information) to the three sets of experiments. During boreal autumn (September-November, SON) and winter, three terms show the most obvious weakening in IV4-2CO_2_ and IV4-4CO_2_. The first one is vertical advection of anomalous temperature by background upwelling [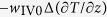; Fig. 4, left two columns], which is caused by the reduced upwelling (Supplementary Fig. S4, *W*) and thermocline fluctuation [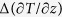]. Due to the reduced background currents (Supplementary Fig. S4, *V*) and meridional temperature gradient changes [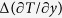], the meridional advection of anomalous temperature by background meridional currents [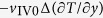; Fig. 4, middle two columns] also weakens remarkably. The last one is zonal advection of background temperature by anomalous zonal currents [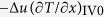; Fig. 4, right two columns], which is responsible for limited westward propagation of the cold SST anomalies (Supplementary Fig. S6). It is clear that both the reduced anomalous easterly and zonal temperature gradient (Supplementary Fig. S4, d_x_SST) are important in inducing the weakening. Note that the cold SST anomaly over the eastern tropical Pacific during summer acts as a trigger for the Bjerknes positive feedback. Once the cooling reduces, oceanic and atmospheric responses will weaken, leading to decreased changes in the thermocline fluctuation [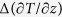] and zonal flow (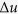).

Thus, the La Niña-like cooling weakens substantially as the CO_2_ concentration increases, due to the El Niño-like change in the tropical Pacific mean state. We show that the El Niño-like warming not only limits the northward migration of the ITCZ, but also reduces the climatological mean currents and zonal SST gradient. The former causes limited SST cooling in the eastern tropical Pacific during boreal summer, and the latter suppresses the strength and extension of the cold SST anomaly through weakened oceanic heat transport. Moreover, the warm SST anomaly over the NTA region decays faster under increased CO_2_ forcing (Figs 2 and 3), because in a warmer climate, both the increased latent heat flux due to enhanced evaporation (Supplementary Fig. S7a) and reduced shortwave radiation caused by a larger amount of cloud cover (Supplementary Fig. S7b) act to damp SST anomalies.

## Discussion

To further demonstrate our results, we discuss changes of the inter-basin teleconnection using 27 models’ outputs included in the fifth phase of the Coupled Model Intercomparison Project (CMIP5). Two sets of climate model simulations are compared, which are the twentieth century historical scenario (HIST) with forcings from the observed changes in the atmospheric composition and the RCP8.5 scenario with a radiative forcing reaching ~8.5 Wm^−2^ near year 2100 (where RCP stands for Representative Concentration Pathway). As evidenced in our analysis, the SST mean state plays an important role in the development of the SST anomalies over the tropical Pacific. We introduce a skill score^21^ for performance of CGCMs in simulating the climatological mean (1900–2005) SST of the tropical Pacific and Atlantic with respect to HadISST (Methods). Fourteen CMIP5 CGCMs are found to be the best, with skill scores higher than the mean of the 27 models (Supplementary Table S2). However, only four out of the 14 models could reproduce a similar impact of the NTA SST anomaly on the tropical Pacific as that in HadISST (Fig. 1). Three of the four models show a weakened response associated with the El Niño-like warming mean state under RCP8.5 (Supplementary Fig. S8–S10), supporting the results of our intermediate climate model. Regardless of model selection, the conclusion still holds. According to Supplementary Table S3, nine of the total 27 models could reasonably simulate the tropical Atlantic-Pacific teleconnection. Among the nine models, four (CanESM2, CCSM4, HadGEM2-ES, INM-CM4) produced weakened response with El Niño-like change in the tropical Pacific mean state, and two (FGOALS-g2, GFDL-ESM2M) produced enhanced response with La Niña-like warming over the tropical Pacific in RCP8.5 runs. As ocean dynamics are reverse under El Niño-like and La Niña-like warming conditions, the above six models actually indicate the same phenomenon and the latter two models demonstrate our conclusion from the opposite perspective. Overall, six of the nine models support the conclusion that global warming will attenuate the tropical Atlantic-Pacific teleconnection under El Niño-like warming of the tropical Pacific. Likewise, an enhanced response might be expected in model simulations if La Niña-like warming trend emerged. It’s clear that efforts are still needed to improve model performance in the simulation of the tropical ocean mean state and climate, as most of the CMIP5 CGCMs fail in reproducing the robust response of the tropical Pacific to the NTA SST anomaly during the twentieth century.

Due to the collective effect of individual interannual events, the teleconnection at decadal timescales might also be expected to weaken under greenhouse warming. This hypothesis is tested by comparing 20-yr running linear trends of SST over the NTA and the east tropical Pacific (Supplementary Table S4; see Supplementary Information for more details). Most models that simulate a weakened (enhanced) response at interannual timescales also produce fewer (more) teleconnection events at decadal timescales, defined as having concurrent trends. Moreover, both the CAM3.1-RGO model and the majority of CMIP5 models (16 of the 27 models) exhibit weakened decadal correlations between the NTA and the tropical Pacific under higher greenhouse gases forcing. However, it is not clear whether the interannual and decadal teleconnections as well as their changes is similar in terms of dynamic processes and mechanisms. Experiments with decadal NTA SST anomalies prescribed are necessary to further confirm the decadal responses in the subsequent studies. Further, the Interdacadal Pacific Oscillation (IPO)^22^ is shown to influence the recent La Niña-like cooling over the tropical Pacific^3^,^23^. Yet how the IPO is involved in the tropical inter-basin teleconnection at different timescales is still unclear.

Except for the NTA SST anomaly in boreal spring, the Atlantic Niño^24^ in boreal summer is also found to impact ENSO through atmospheric teleconnection^25^,^26^,^27^. Previous studies have shown that the former is more responsible for the development of central Pacific El Niño^28^,^29^ events and the latter for eastern Pacific ones^10^,^11^. Other differences include the feature that the Atlantic Niño-Pacific teleconnection seems to have strong decadal variability in association with the Atlantic Multidecadal Oscillation (AMO)^27^. Although we focus on the Atlantic-Pacific teleconnection in the present study, variations in the Indian Ocean may play a role in the tropical Pacific climate variability^30^. It could be interesting and useful to discuss multiscale changes of the Atlantic Niño-Pacific and Indian Ocean-Pacific teleconnections under global warming in further studies.

Our study suggests that greenhouse warming will attenuate the teleconnection from the tropical Atlantic to the Pacific in the future. That is, as greenhouse warming continues, we will not only see less-frequent occurrences of a warming trend interrupted, but also expect weakened impact of the NTA SST anomaly on the ENSO development because of this weakened teleconnection.

## Methods

### Observational data

The observed SST data covering the 1900–2012 period are obtained from Hadley Centre Sea Ice and Sea Surface Temperature data set (HadISST; http://www.metoffice.gov.uk/hadobs/hadisst/)^31^. The monthly mean wind and precipitation data during 1900–2012 are obtained from the ensemble mean fields of the Twentieth Century Reanalysis, version 2 (20CRv2; http://www.esrl.noaa.gov/psd/data/gridded/data.20thC_ReanV2.html)^32^. Note that HadISST is used as the prescribed boundary conditions for the atmospheric model of 20CRv2.

### Model experiments

We conducted three long-time integrated simulations, including a 500-yr control run (CTRL), a 400-yr doubled CO_2_ concentration run (2CO_2_) and a 400-yr quadrupled CO_2_ concentration run (4CO_2_). The last two start from the one hundredth year of CTRL with a sudden doubling (710 ppm) or quadrupling (1420 ppm) of CO_2_ concentration. Note that our analyses are based on the equilibrium states (last 100 years) of the three experiments. Then, based on the respective equilibrium state of CTRL, 2CO_2_ and 4CO_2_, three sets of initial-value ensemble runs were carried out and these are referred to as IV4-CTRL, IV4-2CO_2_ and IV4-4CO_2_. For each member of the experiments, a uniform SST anomaly with amplitude of +4 °C is initiated over the NTA (0°–25°N) region on the first day of January and tracked for two years within the fully coupled environment. A total of 30-member ensemble experiments were performed for each set, with each member starting from a slightly different initial condition. We also carried out three parallel sets of 30 member ensemble experiments, where zero NTA anomalies were initiated (IV0-CTRL, IV0-2CO_2_ and IV0-4CO_2_). The ensemble mean differences between the corresponding sets of experiments are the responses to the imposed NTA SST anomalies.

### CMIP5 models

We analyzed the historical simulations (HIST) over the period 1900–2005 and RCP8.5 experiments over the period 2006–2100, respectively; the former runs are forced by the observed atmospheric composition in the twentieth century and the latter experiments by an escalating radiative forcing throughout the twenty-first century (reaching approximately 8.5 Wm^−2^ in 2100). Supplementary Table S1 lists the 27 CMIP5 models employed in this study and more detailed information can be obtained from http://cmip-pcmdi.llnl.gov/cmip5/availability.html.

### Skill score

We selected CMIP5 models that are more realistic in simulating SST mean state of the tropical Pacific and Atlantic (30°S–30°N, 110°E–20°E) by calculating skill scores (S). As suggested by ref. 21, S = 4(1 + R)^4^/[(SDR + 1/SDR)^2^(1 + R_0_)^4^], where SDR and R denote ratio of standard deviations, and pattern correlation coefficients between CMIP5 and HadISST data, respectively. R_0_ equals 1 for one ensemble member. We calculate SDR and R for each model HIST run against HadISST over the period of 1900–2005 (Supplementary Table S2).

### Regression

We applied lagged regressions between NTA SST anomalies during January to March (JFM) and SST anomalies in the following year to the observational and modeling data^10^,^11^, after removing the linear trend. Note that the impact of ENSO is excluded first by linear regressions with respect to NINO3.4 SST (5°S–5°N, 170°–120°W) during December to February (DJF). All regression results are weighted by the corresponding NTA regional averaged SST anomalies during JFM in order to ensure comparability among different data or scenarios.

## Additional Information

**How to cite this article**: Jia, F. *et al.* Global Warming Attenuates the Tropical Atlantic-Pacific Teleconnection. *Sci. Rep.* **6**, 20078; doi: 10.1038/srep20078 (2016).

## Supplementary Material

Supplementary Information
