# Supplementary material for: Global Warming Attenuates the Tropical Atlantic-Pacific Teleconnection
Source: Sci Rep. 2016 Feb 3;6:20078. doi: 10.1038/srep20078 (PMC4738287; doi:10.1038/srep20078)
Supplement: Supplementary Information [file srep20078-s1.pdf]

**Supplementary Information for**

# Global Warming Attenuates the Tropical Atlantic-Pacific Teleconnection

Fan Jia[1], Lixin Wu[2*], Bolan Gan[2] and Wenju Cai[2,3]

[1] *Institute of Oceanology, Chinese Academy of Sciences, and Key Laboratory of Ocean Circulation and Wave, Chinese Academy of Sciences, Qingdao, China*

[2] *Physical Oceanography Laboratory, Ocean University of China, Qingdao, China*

[3] *CSIRO Oceans and Atmosphere Flagship, Aspendale, Victoria 3195, Australia*

## CAM3.1-RGO Model

The model we used is a fully coupled system consisting of the Community Atmosphere Model version 3.1 (CAM3.1; ref. 1) and a 1.5-layer reduced-gravity ocean (RGO) model with flux corrections[2-5]. The atmospheric component is part of the Community Climate System Model version 3 (CCSM3) developed at the National Center for Atmospheric Research (NCAR; ref. 6). It is based on an Eulerian spectral dynamical core, with a T42 horizontal resolution and 26 vertical levels. The land surface processes in CAM3.1 are represented by the Community Land Model version 3 (CLM3; refs. 7, 8), a fully interactive land model. The oceanic component is an extended Zebiak-Cane type of 1.5-layer RGO model (refs. 9, 10), in which the active upper layer is divided into a fixed depth mixed layer to simulate SST variation and a subsurface layer to parameterize the entrained subsurface temperature through the multivariate linear relationship with thermocline depth. The ocean model covers a global domain (80 °S-80 °N, 0 °-360 °) with 1 °latitude by 2 °longitude resolution, which contains variability off the equatorial band (see more details in refs. 2). It should be noted that the oceanic and atmospheric responses of the tropical Pacific to higher $CO_2$ forcing do not change if different subsurface temperature parameterizations are used[5]. Further, the present model could not reproduce the Subtropical Cell (STC) adjustments and future studies are needed to discuss the role

of STC in the tropical Atlantic-Pacific teleconnection.

**Latent heat flux decomposition**

Based on the standard bulk formula, the latent heat flux (LHF) is calculated as (see refs. 5, 11 for more details):

$$Q_E = L_V C_E \rho_a W (1 - \mathrm{RH} e^{\partial \Delta T}) q_s(T) \quad (1)$$

where $Q_E$ is the LHF, $L_V$ is the latent heat of evaporation, $C_E$ is the transfer coefficient, $\rho_a$ is the surface air density, $W$ is the surface wind speed, RH is the surface relative humidity, $\partial = L_V / (\mathrm{R}_V \mathrm{T}^2) = \sim 0.06\ \mathrm{K}^{-1}$, $\Delta T = T_a - T$, $T_a$ is the surface air temperature, $T$ is the sea surface temperature, and $q_s$ is the saturation specific humidity. By linearizing equation (1), we decompose the LHF into atmospheric forcing and oceanic response (contributions from RH and $\Delta T$ are ignored in this study because they are less important):

$$Q_E^{'} \approx Q_{EO} + Q_{EW} \quad (2)$$

$$Q_{EO} = \frac{\partial Q_E}{\partial T} T^{'} = \partial \overline{Q_E} T^{'} \quad (3)$$

$$Q_{EW} = \frac{\partial Q_E}{\partial W} W^{'} = \frac{\overline{Q_E}}{\overline{W}} W^{'} \quad (4)$$

where the overbar and prime denote the time mean and departure from the mean, respectively. $Q_{EO}$ represents Newtonian cooling because of evaporation and $Q_{EW}$ represents atmospheric forcing because of changes in wind speed (commonly known as the wind-evaporative-SST feedback). In order to diagnose LHF changes in the IV4 experiments, the modeling data are recombined following Jia and Wu[5]. For each ensemble member, the 2-yr IV0 data were added in front of the corresponding 2-yr IV4 data to compose a 4-yr long one. We then applied equation (3) and (4) to the new time series over the northern pole of the SST dipole (5°N-20°N, 140°W-95°W) and defined changes of $Q_{EO}$ and $Q_{EW}$ as the differences between the last and first 2 years of the 4-yr period.

**Ocean heat transport**

Based on the mixed layer heat budget, changes in the ocean heat transport can be decomposed into:

$$\Delta D_o = -\mathbf{U}_{\mathrm{IV0}} \cdot \Delta(\nabla T) - \Delta\mathbf{U} \cdot (\nabla T)_{\mathrm{IV0}} - \Delta\mathbf{U} \cdot \Delta(\nabla T) \qquad (5)$$

where $D_o$ is the ocean heat transport, ***U*** is the three dimensional currents, $T$ is the mixed layer temperature (i.e., SST in our model) and Δ represents changes between the IV4 and IV0 results. We applied equation (5) to the three sets of experiments and analyzed which terms dominate.

**Decadal variability of the tropical Atlantic-Pacific teleconnection**

In order to study decadal variability of the tropical Atlantic-Pacific teleconnection, we computed the linear trend of SST over the NTA and the east tropical Pacific (ETP; 20 °S-20 °N, 180 °-90 °W) based on 20-yr running periods using different datasets. The 20-yr window moves forward starting at every month from 1911 to 2005 in HadISST and CMIP5 HIST runs, 0001 to 0400 in CAM3.1-RGO CTRL ($2CO_2$ and $4CO_2$) runs, and 2006 to 2100 in CMIP5 RCP8.5 runs. Note that the long-term linear trend and seasonal cycle were removed first before calculating the 20-yr running linear trends. In addition, monthly-mean data were used instead of annual-mean values to improve statistical reliability, and only the trends above the 95% confidence level (based on a Student's *t* test) were considered. The cases that have opposite trends emerging in the NTA and the ETP simultaneously were then considered as the decadal-scale teleconnection events. The frequency of these decadal-scale teleconnection events are listed in Table S4.

There are 11.5% of all the overlapped events detected in HadISST exhibit the decadal Atlantic-Pacific teleconnection, most of which occurred during the recent decade (global warming hiatus). However, only a few CMIP5 models could reproduce a similar percentage in the historical runs and almost none of these events occurred after 1990 (not shown). Moreover, most models that produce weakened (enhanced)

responses at interannual timescales also have less (more) teleconnection events at decadal timescale (see Table S3 and models marked with colors in Table S4), indicating that the net effect of individual events over a decadal period may result in overall changes at decadal timescales. Regardless of interannual responses, both the CAM3.1-RGO model and the majority of CMIP5 models (16 of the 27 models) show less-frequent occurrences of the tropical Atlantic-Pacific teleconnection at decadal timescales due to greenhouse warming (the conclusion will not change if 10-yr, 15-yr or 25-yr running window is used). Although the interannual and decadal changes correspond well in our analysis, the dynamic processes and mechanisms may be different. Future studies are still needed to further confirm the decadal responses in detail.

**Table S1 | CMIP5 models used in this study.** Names of models, the associated institution and countries.

| Model name | Institute | Country |
|---|---|---|
| 1. ACCESS1.0 | Commonwealth Scientific and Industrial Research Organization/Bureau of Meteorology | Australia |
| 2. BCC-CSM1.1 | Beijing Climate Center, China Meteorological Administration | China |
| 3. CanESM2 | Canadian Centre for Climate Modelling and Analysis | Canada |
| 4. CCSM4 | National Center for Atmospheric Research | United States |
| 5. CESM1-WACCM | | |
| 6. CMCC-CMS | Centro Euro-Mediterraneo per I Cambiamenti Climatici | Germany |
| 7. CMCC-CM | | |
| 8. CNRM-CM5 | Météo-France/Centre National de Recherches Météorologiques | France |
| 9. CSIRO-MK-3.6.0 | Commonwealth Scientific and Industrial Research Organisation in collaboration with the Queensland Climate Change Centre of Excellence | Australia |
| 10. EC-EARTH | EC-EARTH consortium | Europe |
| 11. FGOALS-g2 | The First Institute of Oceanography, SOA | China |
| 12. GFDL-CM3 | National Oceanic and Atmospheric Administration/Geophysical Fluid Dynamics Laboratory | United States |
| 13. GFDL-ESM2G | | |
| 14. GFDL-ESM2M | | |
| 15. GISS-E2-R | National Aeronautics and Space Administration/Goddard Institute for Space Studies | United States |
| 16. HadGEM2-CC | Met Office Hadley Centre | United Kingdom |
| 17. HadGEM2-ES | | |
| 18. INM-CM4 | Institute for Numerical Mathematics | Russia |
| 19. IPSL-CM5A-MR | Institute Pierre Simon Laplace | France |
| 20. IPSL-CM5A-LR | | |
| 21. IPSL-CM5B-LR | | |
| 22. MIROC5 | University of Tokyo, Atmosphere and Ocean Research Institute; National Institute for Environmental Studies; Japan Agency for Marine Earth Science and Technology | Japan |
| 23. MPI-ESM-LR | Max Planck Institute for Meteorology | Germany |
| 24. MPI-ESM-MR | | |
| 25. MRI-CGCM3 | Meteorological Research Institute | Japan |
| 26. NorESM1-M | Norwegian Climate Centre | Norway |
| 27. NorESM1-ME | | |

**Table S2 | Selection of models.** Criterion used is skill score (S), which is calculated by $S=4(1+R)^4/[(SDR+1/SDR)^2(1+R_0)^4]$. Where SDR and R denotes ratio of standard deviations and pattern correlation coefficients between CMIP5 and HadISST data, respectively. $R_0$ equals 1 for one ensemble member. We applied S to each model HIST run against HadISST during the period of 1900-2005, and over the tropical Pacific and Atlantic region (30 °S-30 °N, 110 °E-20 °E). The multi-model ensemble was evaluated by a simple average of 27 models (MME27). Bold denotes models that have skill scores higher than MME27.

| Model name | SDR | R | S |
|---|---|---|---|
| **1. ACCESS1.0** | **1.003** | **0.931** | **0.869** |
| 2. BCC-CSM1.1 | 0.866 | 0.907 | 0.810 |
| **3. CanESM2** | **1.101** | **0.942** | **0.880** |
| **4. CCSM4** | **0.936** | **0.952** | **0.903** |
| **5. CESM1-WACCM** | **0.946** | **0.918** | **0.843** |
| **6. CMCC-CM** | **1.058** | **0.929** | **0.863** |
| **7. CMCC-CMS** | **1.012** | **0.927** | **0.861** |
| 8. CNRM-CM5 | 0.866 | 0.893 | 0.786 |
| 9. CSIRO-MK-3.6.0 | 1.244 | 0.928 | 0.824 |
| **10. EC-EARTH** | **0.847** | **0.941** | **0.863** |
| 11. FGOALS-g2 | 0.921 | 0.880 | 0.776 |
| **12. GFDL-CM3** | **0.957** | **0.920** | **0.848** |
| 13. GFDL-ESM2G | 0.936 | 0.911 | 0.830 |
| 14. GFDL-ESM2M | 0.900 | 0.891 | 0.790 |
| **15. GISS-E2-R** | **1.035** | **0.911** | **0.833** |
| **16. HadGEM2-CC** | **0.980** | **0.929** | **0.865** |
| **17. HadGEM2-ES** | **0.995** | **0.930** | **0.868** |
| 18. INM-CM4 | 0.972 | 0.904 | 0.821 |
| 19. IPSL-CM5A-LR | 1.131 | 0.908 | 0.816 |
| **20. IPSL-CM5A-MR** | **1.178** | **0.924** | **0.834** |
| 21. IPSL-CM5B-LR | 0.973 | 0.830 | 0.700 |
| 22. MIROC5 | 1.016 | 0.908 | 0.828 |
| **23. MPI-ESM-LR** | **0.958** | **0.924** | **0.855** |
| **24. MPI-ESM-MR** | **0.935** | **0.928** | **0.860** |
| 25. MRI-CGCM3 | 0.933 | 0.871 | 0.762 |
| 26. NorESM1-M | 0.973 | 0.886 | 0.791 |
| 27. NorESM1-ME | 0.987 | 0.891 | 0.800 |
| MME27 | 0.987 | 0.914 | 0.833 |

**Table S3 | Model performance in simulating the tropical Atlantic-Pacific teleconnection and their changes in the RCP8.5 run.** Y (N) in the second column denotes that the model could (could not) reproduce a similar tropical Atlantic-Pacific teleconnection as that in HadISST. The third and fourth columns indicate response of the tropical Atlantic-Pacific teleconnection and warming pattern of the tropical Pacific mean state under RCP8.5, respectively.

| Model name | Validity | Response | Warming Pattern |
|---|---|---|---|
| 1. ACCESS1.0 | N | - | - |
| 2. BCC-CSM1.1 | N | - | - |
| 3. CanESM2 | Y | weakened | El Niño-like |
| 4. CCSM4 | Y | weakened | El Niño-like |
| 5. CESM1-WACCM | N | - | - |
| 6. CMCC-CM | N | - | - |
| 7. CMCC-CMS | Y | enhanced | El Niño-like |
| 8. CNRM-CM5 | N | - | - |
| 9. CSIRO-MK-3.6.0 | N | - | - |
| 10. EC-EARTH | N | - | - |
| 11. FGOALS-g2 | Y | enhanced | La Niña-like |
| 12. GFDL-CM3 | N | - | - |
| 13. GFDL-ESM2G | N | - | - |
| 14. GFDL-ESM2M | Y | enhanced | La Niña-like |
| 15. GISS-E2-R | N | - | - |
| 16. HadGEM2-CC | N | - | - |
| 17. HadGEM2-ES | Y | weakened | El Niño-like |
| 18. INM-CM4 | Y | weakened | El Niño-like |
| 19. IPSL-CM5A-LR | N | - | - |
| 20. IPSL-CM5A-MR | N | - | - |
| 21. IPSL-CM5B-LR | Y | enhanced | El Niño-like |
| 22. MIROC5 | Y | enhanced | El Niño-like |
| 23. MPI-ESM-LR | N | - | - |
| 24. MPI-ESM-MR | N | - | - |
| 25. MRI-CGCM3 | N | - | - |
| 26. NorESM1-M | N | - | - |
| 27. NorESM1-ME | N | - | - |

**Table S4 | Statistics of the tropical Atlantic-Pacific teleconnection at decadal timescale.** We divided the number of events that have opposite trends of SST between the NTA and ETP by all the events that have significant trends in both the NTA and the ETP. Models in blue (red) are the ones that have weakened (enhanced) response at interannual timescales listed in Table S3. The second column shows results derived from HadISST, CAM3.1-RGO CTRL and CMIP5 historical runs. The third columns shows results derived from CAM3.1-RGO $2CO_2$/$4CO_2$ and CMIP5 RCP8.5 runs. Bold denotes models that have less tropical Atlantic-Pacific teleconnection events at decadal time scales in RCP8.5. See text for more details.

| Name | Frequency in HIST | Frequency in RCP8.5 |
|---|---|---|
| HadISST (1911-2005) | 0.115 | - |
| **CAM3.1-RGO** | **0.341 (400-yr CTRL)** | **0.267 (400-yr $2CO_2$)**<br>**0.192 (400-yr $4CO_2$)** |
| 1. ACCESS1.0 | 0.216 | 0.389 |
| **2. BCC-CSM1.1** | **0.085** | **0.015** |
| **3. CanESM2** | **0.149** | **0.023** |
| **4. CCSM4** | **0.007** | **0** |
| **5. CESM1-WACCM** | **0.051** | **0.038** |
| **6. CMCC-CM** | **0.040** | **0** |
| **7. CMCC-CMS** | **0.011** | **0** |
| **8. CNRM-CM5** | **0.021** | **0** |
| 9. CSIRO-MK-3.6.0 | 0.138 | 0.351 |
| **10. EC-EARTH** | **0.054** | **0.007** |
| 11. FGOALS-g2 | 0.202 | 0.290 |
| **12. GFDL-CM3** | **0.058** | **0.036** |
| **13. GFDL-ESM2G** | **0.219** | **0.188** |
| 14. GFDL-ESM2M | 0 | 0.600 |
| 15. GISS-E2-R | 0.039 | 0.794 |
| **16. HadGEM2-CC** | **0.302** | **0.257** |
| **17. HadGEM2-ES** | **0.191** | **0.090** |
| **18. INM-CM4** | **0.016** | **0** |
| **19. IPSL-CM5A-LR** | **0.049** | **0.014** |
| 20. IPSL-CM5A-MR | 0 | 0.032 |
| 21. IPSL-CM5B-LR | 0.179 | 0.221 |
| 22. MIROC5 | 0.107 | 0.190 |
| **23. MPI-ESM-LR** | **0.219** | **0.007** |
| **24. MPI-ESM-MR** | **0.125** | **0.040** |
| 25. MRI-CGCM3 | 0.018 | 0.228 |
| 26. NorESM1-M | 0.013 | 0.091 |
| 27. NorESM1-ME | 0.247 | 0.430 |

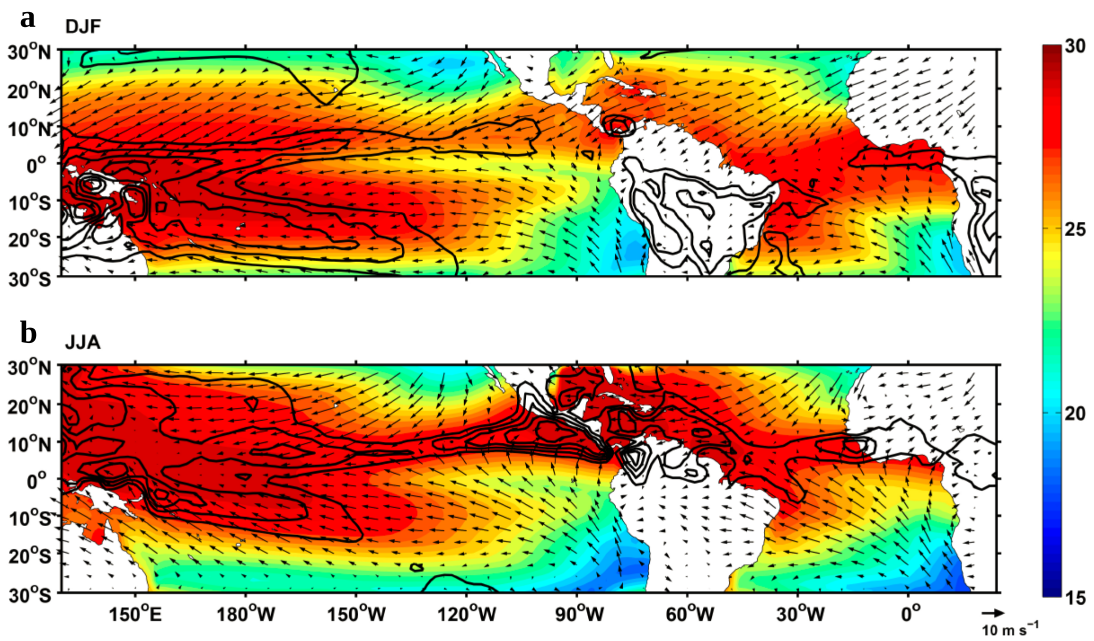


**Figure S1 | Seasonal contrast of climatology. a, b,** DJF and JJA climatological precipitation (black contours at 2 mm $d^{-1}$ intervals; the minimum value for contours is 4 mm $d^{-1}$), SST ( °C; shaded) and wind vector at 10m (m $s^{-1}$; vectors) in model experiment CTRL. All the maps were generated in MATLAB.

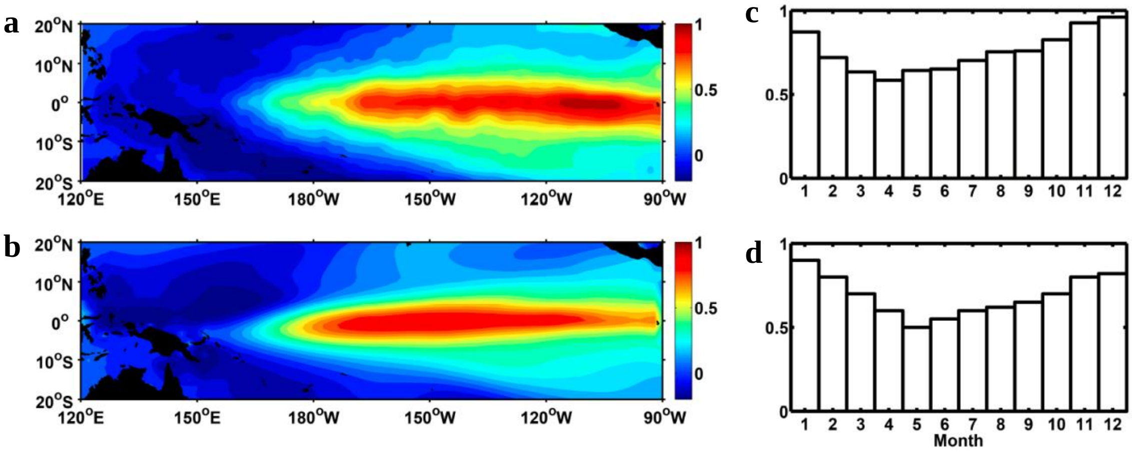


**Figure S2 | Observed and simulated ENSO properties. a, b,** First empirical orthogonal function (EOF1) of SST anomalies derived from the HadISST (year 1901–2000; variance explained: 53.86%), and model CTRL experiment (last 400 year; variance explained: 42.18%), respectively. **c, d,** Standard deviation of Niño3 index (defined as monthly mean SST anomaly in 5 °S–5 °N, 150 °–90 °W) derived from the HadISST and model CTRL, respectively. All the maps were generated in MATLAB.

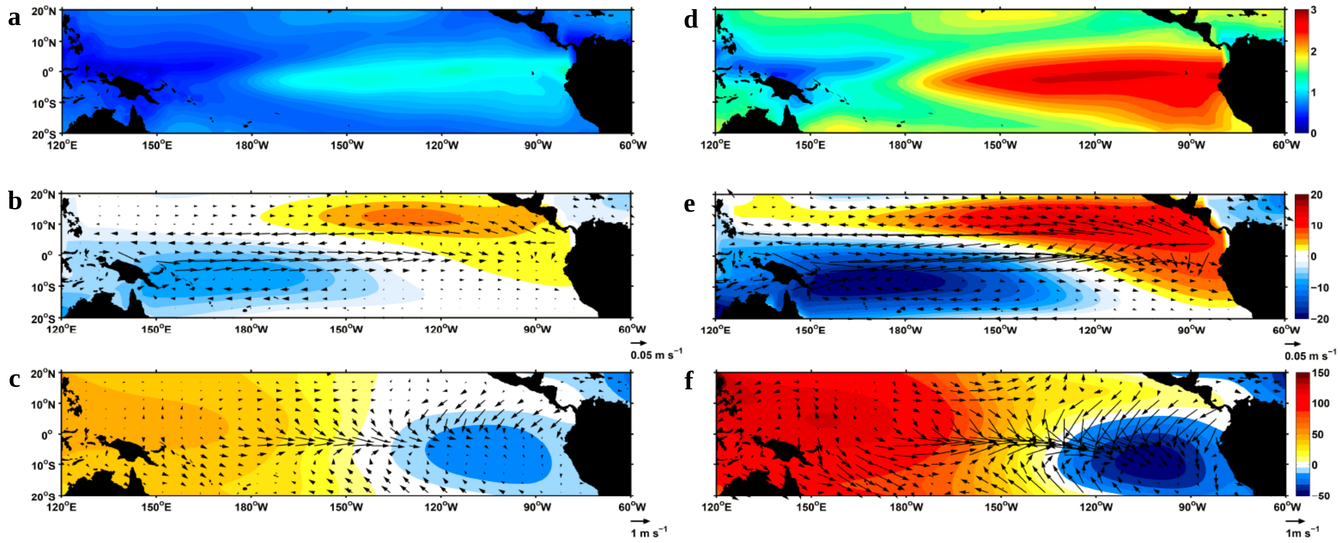


**Figure S3 | El Niño-like response of the tropical Pacific mean states under increasing $CO_2$ forcing**. Changes of the tropical Pacific mean states in the $2CO_2$ run (left column) and $4CO_2$ run (right column) compared with the CTRL experiment, respectively. **a, d,** SST (℃), **b, e,** thermocline depth (m; shaded) and surface current (m $s^{-1}$; vectors), and **c, f,** sea level pressure (Pa; shaded) and surface wind (m $s^{-1}$; vectors). The tropical Pacific mean states indicate climatological mean during the equilibrium state (last 100 years) of the experiments. All the maps were generated in MATLAB.

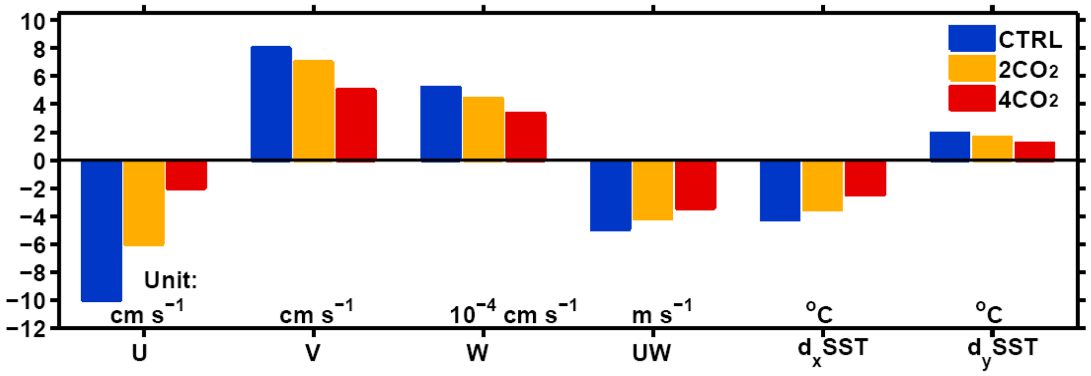


**Figure S4 | Tropical Pacific climatology under different $CO_2$ forcing.** Climatological mean zonal currents (U, V), upwelling (W), surface zonal wind speed (UW), zonal SST gradient ($d_x$SST) and meridional SST gradient ($d_y$SST) in the CTRL (blue bars), $2CO_2$ (orange bars) and $4CO_2$ (red bars) experiments, respectively. The first four values are averaged in the central-eastern Pacific (10$^o$S-10$^o$N, 180$^o$-90$^o$W). The zonal SST gradient is calculated as the SST difference between the eastern (10$^o$S-10$^o$N, 150$^o$W-90$^o$W) and western (10$^o$S-10$^o$N, 135$^o$E-175$^o$E) tropical Pacific. The meridional SST gradient is defined as the SST difference between the east off-equatorial region (5$^o$N-10$^o$N, 150$^o$E-90$^o$W) and the eastern equatorial region (2.5$^o$S-2.5$^o$N, 155$^o$E-120$^o$W).

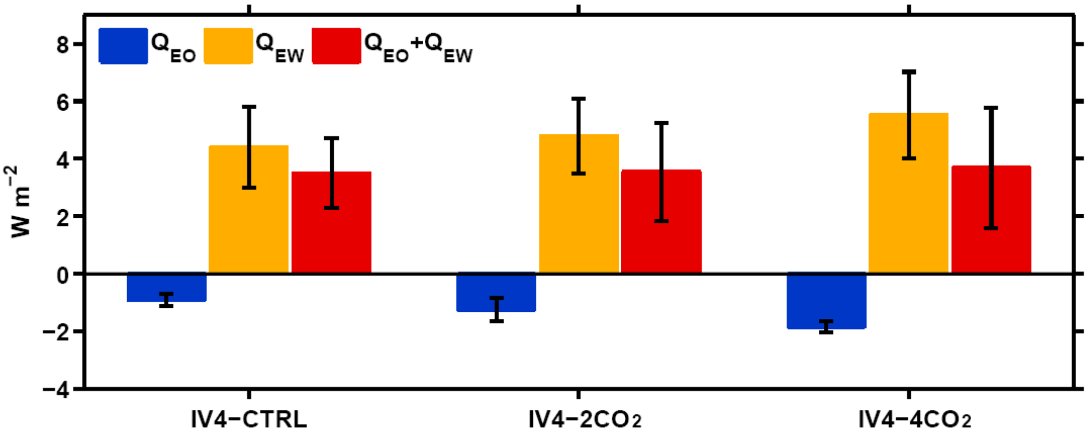


**Figure S5 | Latent heat flux decomposition and their changes under different $CO_2$ forcing.** Ensemble-mean changes of latent heat flux components in the IV4-CTRL (left), IV4-2$CO_2$ (middle) and IV4-4$CO_2$ (right) experiments compared with the corresponding IV0 runs. The decomposition was applied over the northern pole (5°N-20°N, 140°W-95°W; i.e., red box region in Figure 3a) of the SST dipole during MAM season. The blue, orange and red bars denote $Q_{EO}$, $Q_{EW}$ and sum of the two, respectively. Standard deviation bars based on the standard deviation of ensemble members are also shown.

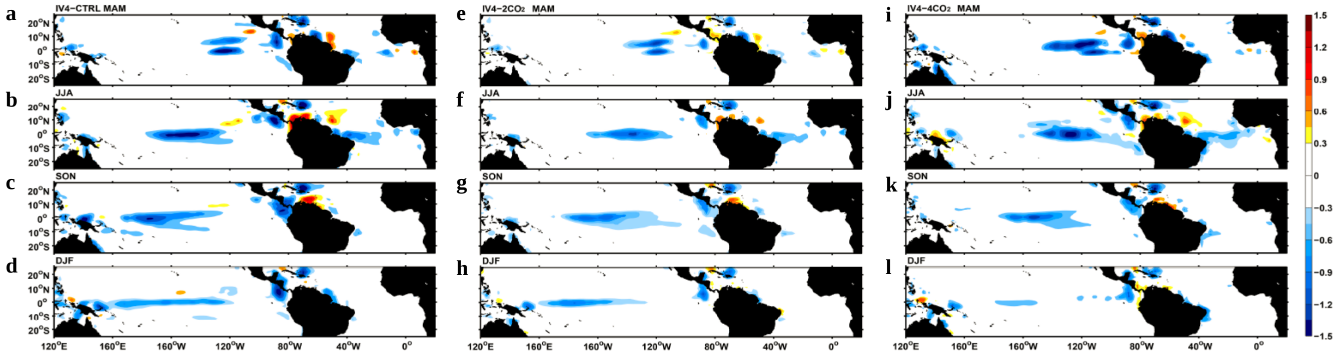


**Figure S6 | Changes of zonal advection of mean temperature by anomalous currents under different $CO_2$ forcing.** Ensemble- and seasonal-mean changes of zonal advection of mean temperature by anomalous zonal currents (unit: $1\times10^{-7}$ ℃ $s^{-1}$; $-\Delta u(\partial T / \partial x)_{IV0}$) in the IV4-CTRL (left; **a-d**), IV4-2$CO_2$ (middle; **e-h**) and IV4-4$CO_2$ (right; **i-l**) experiments compared with the corresponding IV0 runs. Only values above the 95% confidence level are shown. All the maps were generated in MATLAB.

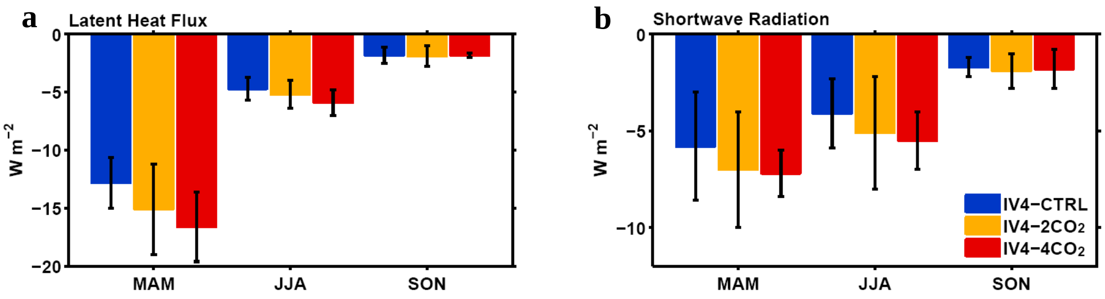


**Figure S7 | Changes of net sea surface latent heat flux and shortwave radiation under different $CO_2$ forcing. a, b,** Ensemble-mean changes of net sea surface latent heat flux and shortwave radiation during MAM, JJA and SON season over the north tropical Atlantic (NTA). The bars denote changes in the IV4-CTRL (blue), IV4-2$CO_2$ (orange) and IV4-4$CO_2$ (red) experiments compared with the corresponding IV0 runs. Standard deviation bars based on the standard deviation of ensemble members are also shown.

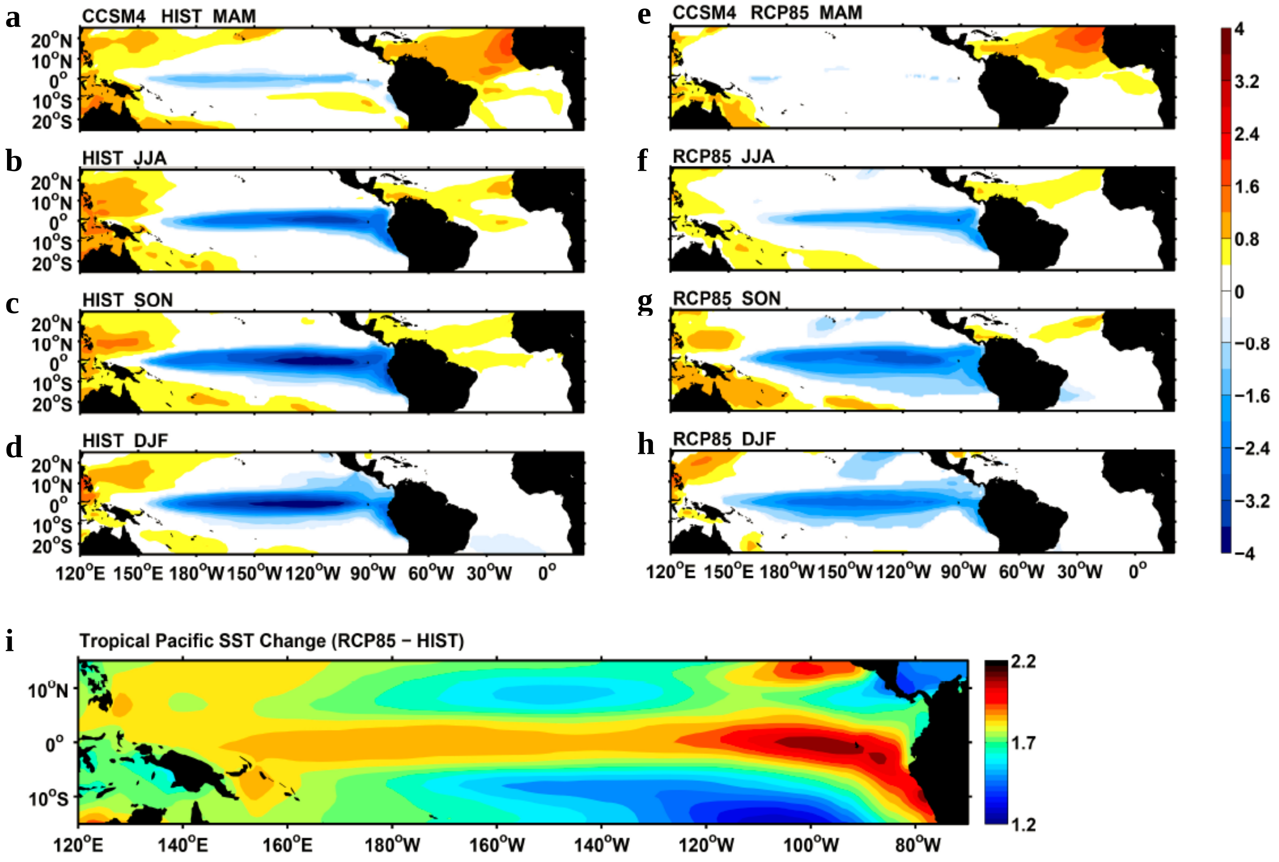


**Figure S8 | Lagged regressions with respect to NTA SST in the RCP8.5 and HIST run as well as tropical Pacific SST changes in the RCP8.5 run.** Lagged regressions between NTA SST (90 °W-20 °E, 0-25 °N) averaged during the January-February-March (JFM) season and SST (°C; shaded) in the following year. The left (**a-d**) and right (**e-h**) columns are derived from HIST (1900-2005) and RCP8.5 (2006-2100) run of CCSM4, respectively. Only values above the 95% confidence level are shown. **i,** Climatological mean changes of the tropical Pacific SST between the RCP8.5 (2006-2100) and the HIST (1900-2005) run. All the maps were generated in MATLAB.

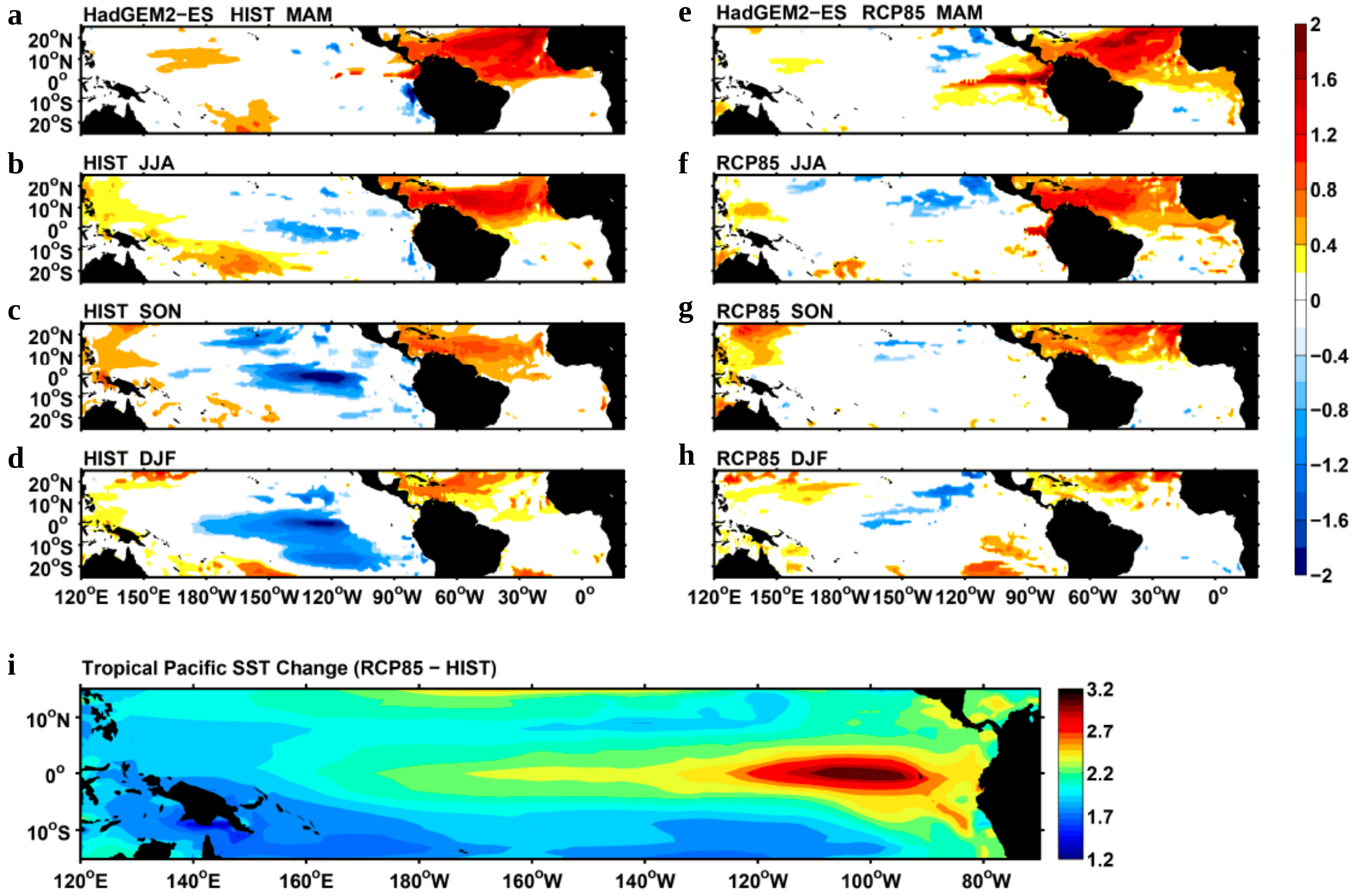


**Figure S9 | Same as those in Figure S8 but derived from HadGEM2-ES outputs.** All the maps were generated in MATLAB.

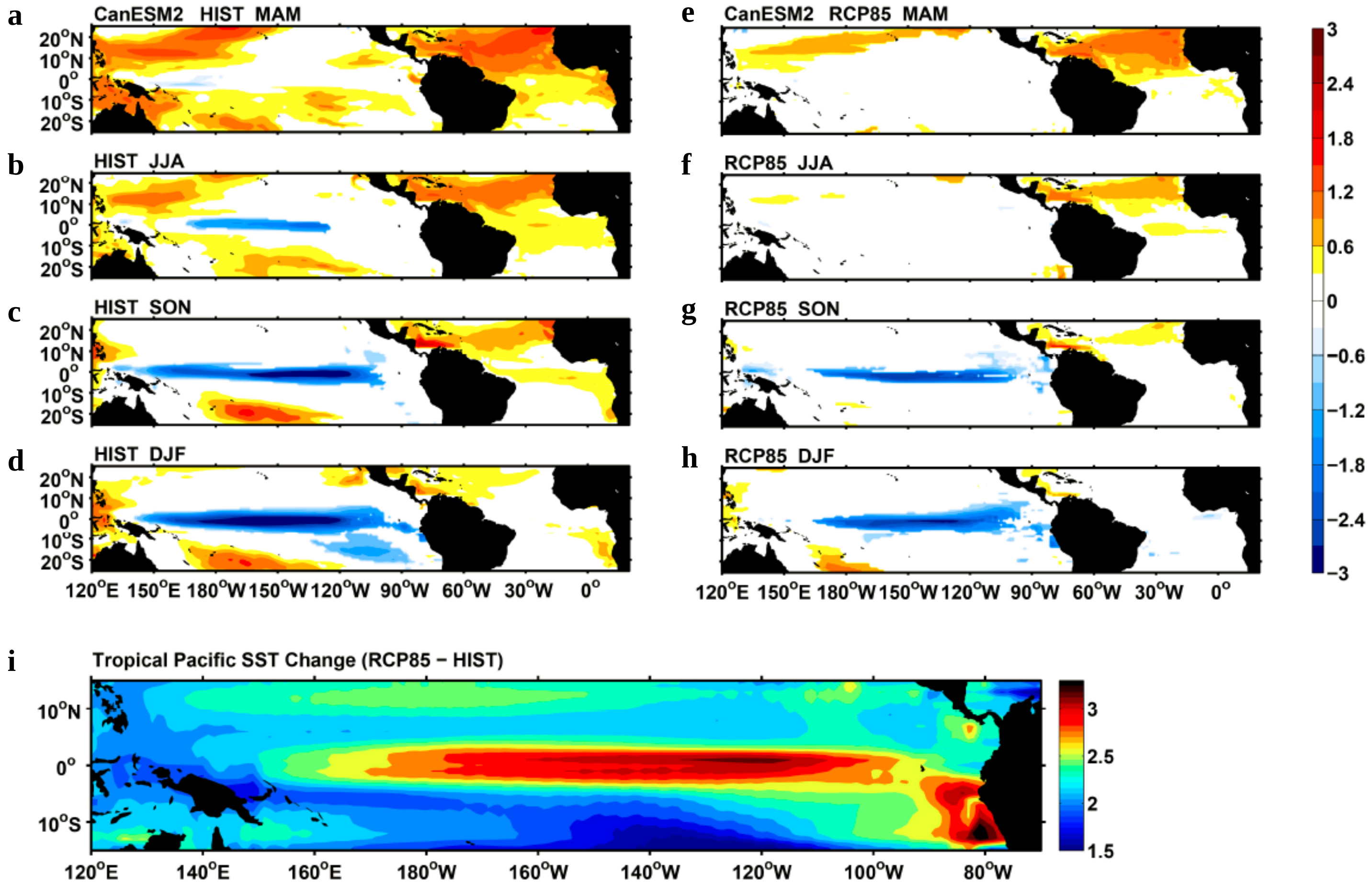


**Figure S10 | Same as those in Figure S8 but derived from CanESM2 outputs.** All the maps were generated in MATLAB.

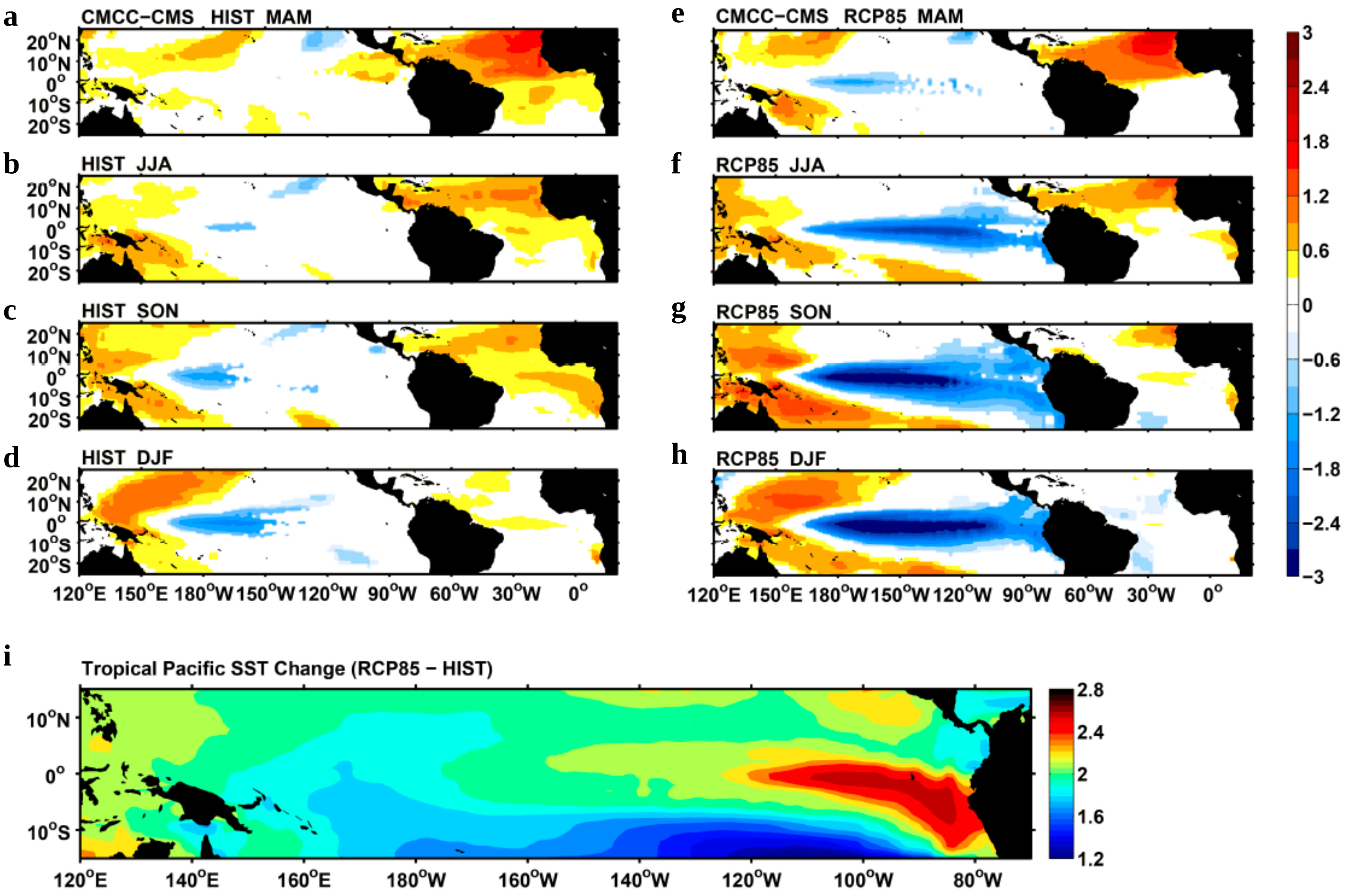


**Figure S11 | Same as those in Figure S8 but derived from CMCC-CMS outputs.** All the maps were generated in MATLAB.

## References


1. Collins, W. D. *et al.* The formulation and atmospheric simulation of the Community Atmospheric Model: CAM3. *J. Clim.* **19**, 2144–2161 (2006).
2. Fang, Y. A coupled model study of the remote influence of ENSO on tropical Atlantic SST variability. *Ph.D. thesis, Texas A&M University*, **93** pp (2005).
3. Chiang, J. C. H., Fang, Y. & Chang, P. Interhemispheric thermal gradient and tropical Pacific climate. *Geophys. Res. Lett.* **35**, L14704 (2008).
4. Zhang, L., Chang, P. & Ji, L. Linking the Pacific meridional mode to ENSO: Coupled model analysis. *J. Climate* **22**, 3488-3505 (2009).
5. Jia, F. & Wu, L. A study of response of the equatorial Pacific SST to doubled-$CO_2$ forcing in the coupled CAM-1.5 layer reduced-gravity ocean model. *J. Phys. Oceanogr.* **43**, 1288–1300 (2013).
6. Collins, W. D. *et al.* The Community Climate System Model, version 3 (CCSM3). *J. Clim.* **19**, 2122–2143 (2006).
7. Bonan, G. B. *et al.* The land surface climatology of the Community Land Model coupled to the NCAR Community Climate Model. *J. Clim.* **15**, 3123–3149 (2002).
8. Oleson, K. W. *et al.* Technical description of the Community Land Model (CLM). NCAR Tech. Note NCAR/TN-461STR, Boulder, CO, 174 (2004).
9. Clement, A. C., Seager, R., Cane, M. A. & Zebiak, S. E. An ocean dynamical thermostat. *J. Clim.* **9**, 2190–2196 (1996).
10. Zebiak, S. E. & Cane, M. A. A model ENSO. *Mon. Wea. Rev.* **115**, 2262–2278 (1987).
11. Richter, I. & Xie, S.-P. The muted precipitation increase in global warming simulations: A surface evaporation perspective. *J. Geophys. Res.* **113**, D24118 (2008).
